# Environmental complexity buffers against stress-induced negative judgement bias in female chickens

**DOI:** 10.1038/s41598-018-23545-6

**Published:** 2018-03-29

**Authors:** Josefina Zidar, Irene Campderrich, Emelie Jansson, Anette Wichman, Svante Winberg, Linda Keeling, Hanne Løvlie

**Affiliations:** 1 Department of Physics, Chemistry and Biology, IFM Biology, Linköping University, SE 581 83, Linköping, Sweden; 2 Department of Animal Environment and Health, Swedish University of Agricultural Sciences, SE 750 07, Uppsala, Sweden; 3 Neiker-Tecnalia, Department of Animal Production, 01080 Vitoria-Gasteiz, Spain; 4 Department of Neuroscience, Uppsala Biomedical Centre BMC, Uppsala University, Box SE 751 24, Uppsala, Sweden

## Abstract

Cognitive processes are often biased by emotions. In humans, affective disorders are accompanied by pessimistic judgement, while optimistic judgement is linked to emotional stability. Similar to humans, animals tend to interpret ambiguous stimuli negatively after experiencing stressful events, although the long-lasting impact on judgement bias has rarely been investigated. We measure judgement bias in female chicks (*Gallus gallus domesticus*) after exposure to cold stress, and before and after exposure to additional unpredictable stressors. Additionally, we explore if brain monoamines can explain differences in judgement bias. Chicks exposed to cold stress did not differ in judgement bias compared to controls, but showed sensitivity to additional stressors by having higher motivation for social reinstatement. Environmental complexity reduced stress-induced negative judgement bias, by maintaining an optimistic bias in individuals housed in complex conditions even after stress exposure. Moreover, judgement bias was related to dopamine turnover rate in mesencephalon, with higher activity in individuals that had a more optimistic response. These results demonstrate that environmental complexity can buffer against negative effects of additive stress and that dopamine relates to judgement bias in chicks. These results reveal that both internal and external factors can mediate emotionally biased judgement in animals, thus showing similarities to findings in humans.

## Introduction

It is well known from human psychology that emotions can affect several aspects of cognition (for review, see refs^1,2^). How people interpret, attend to, and remember information is affected by the individual’s affective state or valence^3^. Individuals in a negative affective state will for example interpret ambiguous stimuli negatively^4^ and likewise anticipate negative future events^5^. In humans, anxiety and depression associate with negative biases (e.g refs^1,2^). Such emotionally biased information processing is termed cognitive judgement bias (e.g. refs^2,3^), judgement bias, or interpretation bias^6^.

In the seminal study by^7^, judgement bias comparable to humans, was observed in rats (*Rattus norvegicus*). Rats were exposed to two different husbandry regimes in an attempt to induce differences in affective state. Rats that had been exposed to an unpredictable environment with randomly occurring negative events, showed a reduced anticipation of positive events when exposed to ambiguous stimuli, compared to controls^7^. Following this study, researchers of animal behaviour and animal welfare have repeatedly demonstrated emotionally biased judgement in a range of species (for review, see refs^3,6,8–11^). A few studies have aimed to induce a positive affective state (e.g. refs^12–14^). For example, in rats, optimistic bias was successfully induced in rats that ‘laughed’ when they were being tickled^14^. More often, studies aim to induce a negative affective state. Pessimistic judgement has most often been the result of such treatments (e.g. refs^7,15–20^), but results are somewhat inconclusive^21–25^.

The methods used to induce differences in affective state include manipulating the environment by for instance removing or introducing complexity, or enrichment (e.g. refs^15,26,27^), applying unpredictability in aversive events (e.g. refs^7,18^), or exposing animals to acute stress, normally by using standard but likely stressful, husbandry regimes (e.g. dehorning^19,20^, shearing^22^, maternal separation^20^). The methods used to introduce variation in affective state and thereby judgement thus ranges from short to long-term treatments and are of varying severity.

Stressful experiences can have profound effects on brain, behaviour and physiology^28,29^. During development, the effects of stress are often enhanced and can be long-lasting^28^. The developing brain is sensitive and undergoes dramatic changes in structure and function^30^. Stress during the juvenile phase can therefore affect neural development^30^, and cause learning deficits^18,31,32^. How stress early in life influences judgement biases in animals is less explored (but see refs^23,33^). In humans, we know that stress early in life can cause affective disorders later in life, in which emotional and cognitive processes are disturbed^34,35^. In rats, juvenile stress and chronic unpredictable stress during adolescence had a long-term effect on judgement bias^23,33^, suggesting that stress early in life can have lasting effects on judgement biases, also in non-human animals.

Brain monoaminergic systems are recognized as possible mediators of neuronal plasticity and cognitive function, and are involved in behavioural mechanisms and processes, including responses to stress and behaviour^36–38^. The monoaminergic systems are therefore candidate modulators of judgement biases. Indeed, recent studies show that enhancing dopaminergic function in the brain (using administration of 3–4,dihydroxy-L-phenylalanine; L-DOPA) increases optimistic judgement in humans^39,40^, and rats^41^. Similarly, a dopamine antagonist (fluphenazine) abolished optimistic judgement observed in bumblebees (*Bombus terrestris audax*) after sucrose consumption^42^, suggesting that at least dopamine is involved in regulating judgement biases in these species. There is still limited knowledge about the impact of brain monoamines on judgement biases in other species, and few studies have looked at un-manipulated levels of these brain monoamines in animals with varying affective state (but see ref.^16^).

Here we aim to test how early aversive events affect judgement bias in young female domestic fowl (*Gallus gallus domesticus*). Additionally, we aim to investigate whether increased environmental complexity can reduce negative effects of stress on affective states. Environmental enrichment can improve learning and memory (pigs, *Sus scrofa*^43,44^), affect adult personality (red junglefowl, *Gallus gallus*^45^), reduce negative effects of stress (e.g. refs^46,47^), and induce optimistic biases (e.g. refs^12,26,27,48,49^). Here we exposed young female domestic fowl housed in more complex and simpler environments to judgement bias tests after exposure to early cold stress (2 days post-hatching) and before and after chicks were exposed to 5 days of unpredictable aversive events (at 4 weeks of age). In short, individuals learned to discriminate between a rewarded (e.g. white) and an unrewarded (e.g. black) colour cue before being presented with the learnt cues as well as three intermediate unknown grey colour cues (presented one at the time). Latency for a chick to reach these colour cues was used to measure variation in judgement bias. A response similar to a rewarded cue is considered an optimistic response, whereas a response similar to an unrewarded cue is considered a less optimistic response^3^. We predict that environmental complexity may attenuate stress-induced negative judgement bias after stress exposure, and thus buffer against negative effects of stress. To explore the potential influence of underlying variation in monoaminergic systems on judgement bias, we performed brain dissections and measured levels and turnover rates of norepinephrine, serotonin and dopamine in various brain areas of test individuals. Based on previous studies on humans, rats and bumblebees^39–42^, we predict that dopamine will influence judgement bias also in the fowl.

## Results

To ascertain that individuals had learnt to associate a colour cue with a reward prior to experiencing ambiguous cues in our judgement bias task, they had to make 6 correct choices in a row in an association task. There was large variation in learning speed among individuals (range: 9–89 trials needed to learn the association), and eighty-seven out of ninety-six individuals learned to associate the colour cue with the reward.

We used the probability for chicks to approach the colour cues presented (i.e. whether a chick approached the cue or not, within 30 seconds) in our first judgement bias task to explore their judgement prior to being exposed to the unpredictable stressors. Chicks approached both the rewarded cue and the close to rewarded cue 98.8% of the time, the middle cue 89% of the time, and the near unrewarded and unrewarded cues 58 and 54% of the time, respectively. The probabilities of approaching ambiguous cues were thus over chance level, guiding our interpretation of responses measured in the judgement bias task as measuring variation in optimistic bias^9,27^.

### Effect of cold stress

**Figure 1:**
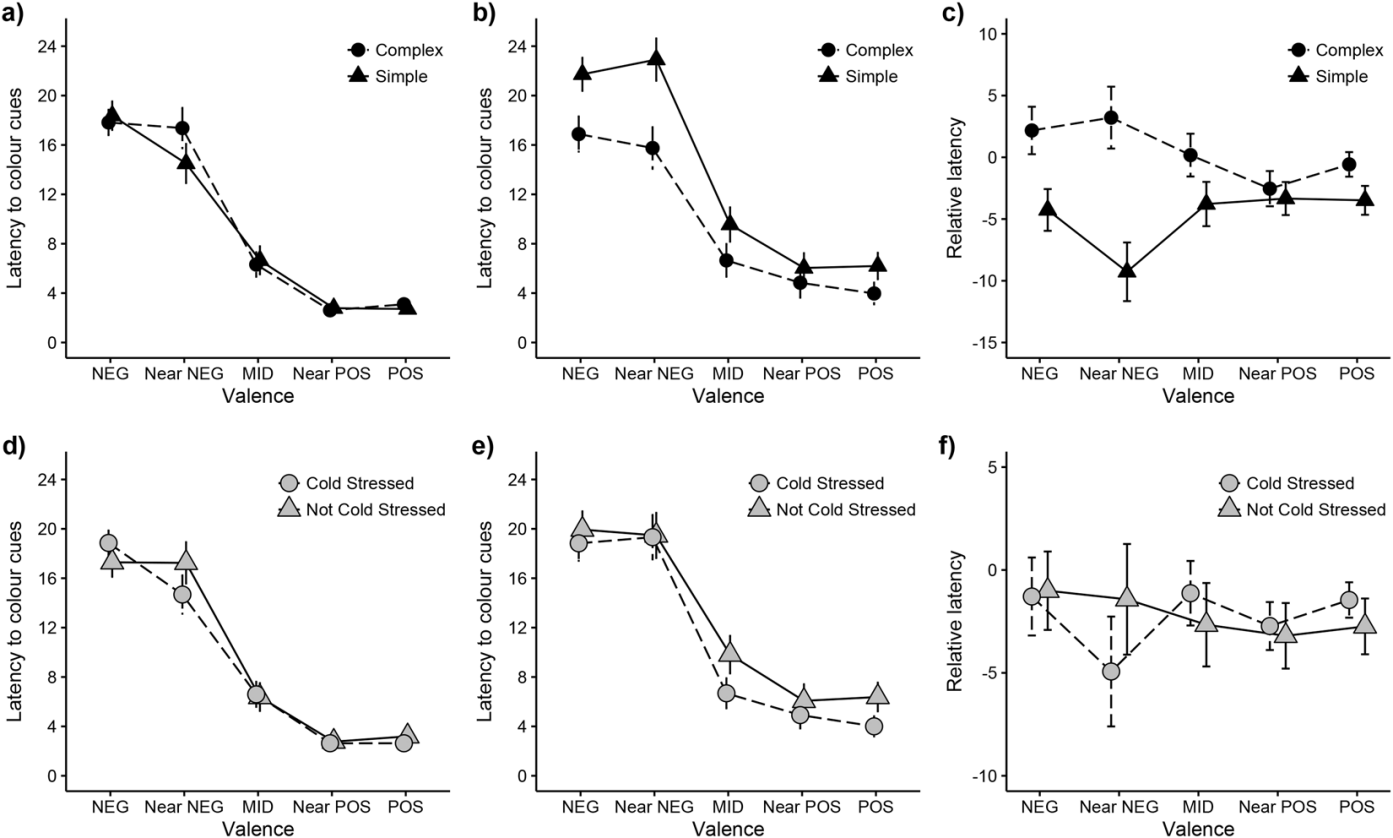
Influence of stress and environmental complexity on judgement bias in young female domestic fowl. Latency in seconds (mean ± SE) to approach colour cues in a judgement bias test in relation to cold stress treatments and environmental complexity before (**a**,**d**) and after (**b**,**e**) chicks were exposed to a battery of unpredictable stressors. Cold stress treatment in young chicks did not affect optimism in chicks before (**d**) or after (**e**) exposure to unpredictable stressors and therefore we did not observe a changed response from the 1^st^ to the 2^nd^ judgement bias test (**f**). Environmental complexity did not affect latency to reach the colour cues before (**a**) exposure to unpredictable stressors, but after (**b**) exposure to unpredictable stressors, chicks in complex conditions had shorter latencies to reach the ‘NEG’, ‘NearNEG’ and ‘NearPOS’ colour cues, showing a changed response from the 1^st^ to the 2^nd^ test (**c**) and were thus less optimistic. ‘NEG’ = unrewarded cue, ‘NearNEG’ = cue close to unrewarded cue, ‘MID’ = intermediate cue between rewarded and unrewarded cue, ‘NearPOS’ = cue close to rewarded cue, ‘POS’ = rewarded cue.

We measured individual chick’s latency (in seconds) to reach presented colour cues in our judgement bias test after exposure to cold stress, and again after exposing them to 5 days of additional unpredictable stressors. Cold stress did not influence latency to the colour cues in the first or the second judgement bias task (p > 0.24 for all cues, and with Bonferroni correction even less significant, Fig. 1d). Confirming this and based on model selection, models best explaining variation in our data did not include the variable ‘Cold stress’ (Table 1). This means that this variable was not very important in our models (Table 2), and show that there were no difference between the cold stressed and not cold stressed birds in responses to the judgement bias test either before or after adding the additional stressors (cold stress: mean 27.69 ± 0.78 sec; not cold stress: mean 27.79 ± 0.87 sec Fig. 1d–f).

**Table 1:** Models explaining variation in judgement bias among young female domestic fowl. Judgement bias was measured as individual change in latency to approach colour cues between two judgement bias tests (for more details, see main text). Models are ranked according to their AICc value and weight (ω), where lower AICc values and ω weight imply a better goodness of fit. Models shown are within ΔAICc <2. For comparison, ‘Null’ models are shown despite having ΔAICc >2. Estimates for all supported variables are given. In cases where several variables are included in a model, variables are presented in the same order as they are presented in the ‘Model column’, separated by a straight line. Estimates (mean ± SE) for categorical explanatory variables are given in the results section.

Model	Rank	Estimate	AICc	ΔAICc	ω
**Individual change in latency to colour cues**					
**(i) Influence of cold stress, environmental complexity, and norepinephrine**					
EC	1	+	2173.30	0.00	0.37
EC | Tel_NE	2	+| 0.01	2174.80	1.50	0.18
EC | Ot_NE	3	+| 0.00	2174.90	1.62	0.17
EC | Cue	4	+| −0.63	2175.10	1.84	0.15
EC | Mes_NE	5	+| 0.00	2175.20	1.95	0.14
Null	24		2178.10	4.79	0.01
**(ii) Influence of cold stress, environmental complexity, and dopamine**					
EC	1	+	2173.30	0.00	0.44
EC | Ht_DA	2	+| −0.08	2174.60	1.32	0.23
EC | Cue	3	+| −0.63	2175.10	1.84	0.17
EC | Mes_DA	4	+| −0.11	2175.20	1.93	0.17
Null	24		2178.10	4.79	0.00
**(iii) Influence of cold stress, environmental complexity, and serotonin**					
EC	1	+	2173.30	0.00	0.45
EC | Ht_5-HT	2	+| 0.00	2174.90	1.66	0.20
EC | Cue	3	+| −0.63	2175.10	1.84	0.18
EC | Tel_5-HT	4	+| 0.00	2175.20	1.93	0.17
Null	24		2178.10	4.79	0.00
**(iv) Influence of cold stress, environmental complexity, and dopamine turnover rate**					
EC | Mes_DOPAC/DA	1	+| 5.85	2171.80	0.00	0.36
EC | Mes_DOPAC/DA | Ot_DOPAC/DA	2	+| 6.65 | −0.35	2173.10	1.31	0.19
EC	3	+	2173.30	1.50	0.17
EC | Mes_DOPAC/DA | Cue	4	+| 5.85 | −0.63	2173.60	1.86	0.14
EC | Mes_DOPAC/DA | Ht_DOPAC/DA	5	+| 5.97 | −0.41	2173.70	1.89	0.14
Null	50		2178.10	6.29	0.00
**(v) Influence of cold stress, environmental complexity, and serotonin turnover rate**					
EC	1	+	2173.30	0.00	0.36
EC, Tel_5-HIAA/5-HT	2	+| −71.51	2174.70	1.41	0.18
EC, HT_5-HIAA/5-HT	3	+| −7.55	2174.70	1.42	0.18
EC, Cue	4	+| −0.63	2175.10	1.84	0.14
EC, Mes_5-HIAA/5-HT	5	+|−13.79	2175.10	1.88	0.14
Null	24		2178.10	4.79	0.00

CS – Cold stress, EC – Environmental complexity, Cue – Colour cues, NE – Norepinephrine, DA – Dopamine, 5-HT – Serotonin, DOPAC/DA – Dopamine turnover rate, 5-HIAA/5-HT – Serotonin turnover rate, Ht – Hypothalamus/thalamus, Tel – Telencephalon, Mes – Mesencephalon, Ot – Optic tectum.

**Table 2:** Overview of importance of variables from the selected models explaining judgement bias among young female domestic fowl. The sum of AICω (**Σ**AICω) for variables occurring in supported models (Table 2, obtained from models with accumulative weight 0.95). **Σ**AICω close to 1 show variables that are included in all of the supported models and, conversely, towards 0 if the variables appear in few supported models.

Model	ΣAICω
**i) Influence of cold stress, environmental complexity, and norepinephrine**	
EC	1.00
Tel_NE	0.20
Ot_NE	0.19
Cue	0.17
**(ii) Influence of cold stress, environmental complexity, and dopamine**	
EC	1.00
Ht_DA	0.27
Cue	0.21
**(iii) Influence of serotonin**	
EC	1.00
Ht_5-HT	0.24
Cue	0.22
**(iv) Influence of cold stress, environmental complexity, and dopamine turnover rate**	
EC	1.00
Mes_DOPAC/DA	0.80
Ot_DOPAC/DA	0.22
Cue	0.17
**(v) Influence of cold stress, environmental complexity, and serotonin turnover rate**	
EC	1.00
Tel_5-HIAA/5-HT	0.21
Ht_5-HIAA/5-HT	0.21
Cue	0.17

CS – Cold stress, EC – Environmental complexity, Cue – Colour cues, NE – Norepinephrine, DA – Dopamine, 5-HT – Serotonin, DOPAC/DA – Dopamine turnover rate, 5-HIAA/5-HT – Serotonin turnover rate, Ht – Hypothalamus/thalamus, Tel – Telencephalon, Mes – Mesencephalon, Ot – Optic tectum.

After exposure to cold stress and both before and after exposing birds to unpredictable stressors, we also measured the behaviour of chicks in a multivariate behaviour test to investigate social, cognitive and risk-taking behaviour (Figure S4a,b). The latency until a chick started moving was not affected by exposure to cold stress early in life (Table 3). However, after the additional exposure of unpredictable stressors, individuals that had been exposed to cold stress had shorter latencies to start moving (mean = 8.76 ± 2.81 sec; Table 3) compared to not cold stressed chicks (mean = 18.88 ± 4.97 sec; Table 3). Latency to solve a detour in the multivariate behaviour test was not affected by any of the stressors (Table 3). The amount of time chicks spent in three zones of the arena (i.e. ‘Inner circle’ - time spent close to pen mates; ‘Outer circle’ - time spent further away from pen mates but within visual contact; ‘Behind screen’ - time spent further away and without visual contact with pen mates), was not affected by cold stress or the combination of cold stress and the additional stressor (Table 3, see SI Figure S4 for details on the zones). Cold stress early in life did not influence any of the brain monoamines examined (Table 4).

**Table 3:** Generalized linear mixed models (GLMM) of the effect of early cold stress and environmental complexity on behaviour in a multivariate behavioural assay in young female domestic fowl. Test 1 is before and test 2 after adding a week of unpredictable stress.

	χ^2^	df	p
*Before additional stressor (test 1)*			
**Latency to move**			
EC	0.05	1	0.82
CS	0.26	1	0.61
EC × CS	0.18	1	0.67
**Latency to find entrance**			
EC	0.86	1	0.35
CS	0.94	1	0.33
EC × CS	0.08	1	0.78
*After exposure to additional stressor (test 2)*			
**Latency to move**			
EC	4.49	1	0.03**
CS	5.34	1	0.02**
EC × CS	0.68	1	0.41
**Latency to find entrance**			
EC	4.46	1	0.03**
CS	1.61	1	0.20
EC × CS	0.00	1	1.00
**Time spent close to their conspecifics (Inner circle)**,			
EC	5.14	1	0.02**
CS	0.29	1	0.59
EC × CS	0.00	1	0.97
**Time spent with visual contact with conspecifics but not close to them (Outer circle)**			
EC	2.00	1	0.16
CS	0.01	1	0.93
EC × CS	0.14	1	0.29
**Time spent without visual contact with conspecifics (Behind screen)**			
EC	7.22	1	0.007***
CS	1.09	1	0.30
EC × CS	0.86	1	0.35

CS – Cold stress, EC – Environmental complexity. Statistical significance is indicated by asterisk symbols: *P < 0.05 level, **P < 0.01, ***P < 0.001.

**Table 4:** Variation in brain monoamines explained by Cold Stress and Environmental Complexity.

	Cold Stress			Environmental Complexity		
	χ^2^	df	p	χ^2^	df	p
**Norepinephrine**						
Ht	0.53	1	0.47	2.18	1	0.14
Tel	0.62	1	0.43	1.05	1	0.31
Mes	1.74	1	0.27	1.62	1	0.20
Ot	1.52	1	0.22	0.97	1	0.33
**Dopamine**						
Ht	0.00	1	0.99	0.39	1	0.53
Tel	0.87	1	0.35	2.08	1	0.15
Mes	3.49	1	0.06	0.56	1	0.45
Ot	3.68	1	0.06	1.23	1	0.27
**Serotonin**						
Ht	2.52	1	0.11	1.21	1	0.27
Tel	0.85	1	0.36	2.13	1	0.15
Mes	1.49	1	0.22	2.16	1	0.14
Ot	1.56	1	0.21	0.62	1	0.43
**Dopamine turnover rate**						
Ht	1.21	1	0.27	0.38	1	0.54
Tel	0.11	1	0.74	0.20	1	0.65
Mes	0.38	1	0.54	0.00	1	0.97
Ot	3.07	1	0.08	1.12	1	0.29
**Serotonin turnover rate**						
Ht	0.05	1	0.83	0.13	1	0.72
Tel	0.09	1	0.77	0.01	1	0.93
Mes	3.20	1	0.07	2.65	1	0.10
Ot	1.33	1	0.24	2.38	1	0.12

Ht – Hypothalamus/thalamus, Tel – Telencephalon, Mes – Mesencephalon, Ot – Optic tectum.

### Effect of environmental conditions

There was no difference in how individuals from the simpler and more complex environment responded to the judgement bias test (i.e. latency to reach the colour cues) prior to exposure of unpredictable stressors (p > 0.07 for all cues, and with Bonferroni correction even less significant, Fig. 1a).

On the other hand, analyses of the alteration of response from the first to the second judgement bias test, model selection showed that after a period of unpredictable stressors, how individuals were housed altered their response in the second judgement bias test (complex: mean 30.49 ± 0.81 sec; simple: mean 25.17 ± 0.78 sec; Table 1, Fig. 1b,c). This is shown by that the variable ‘Environmental condition’ is always appearing in top models (Table 1) and show high importance in these models (Table 2). Further analyses show that individuals from simpler environments significantly changed their responses after exposure to additional stressors compared to before the stressors were applied. The changed response was observed toward the unrewarded negative cue and the ambiguous near negative and near positive cues (comparing responses in 1^st^ vs 2^nd^ judgement bias test: NearPOS: W = 70, p = 0.01, NearNEG: W = 97, p = 0.01, NEG: W = 70, p = 0.001), but not toward the other cues (POS: W = 119, p = 0.10, MID: W = 136, p = 0.14), while individuals from more complex environments did not change their response to any of the cues (POS: W = 250, p = 1.00, NearPOS: W = 240, p = 1.00, MID: W = 265, p = 1.00, NearNEG: W = 148, p = 1.00, NEG: W = 227, p = 1.00).

There was no significant difference in latency to move or latency to find the entrance in the multivariate behavioural test preceding the unpredictable stress between individuals in more complex or simpler conditions (Table 3). However, after exposure to unpredictable stress, individuals housed in more complex conditions had shorter latencies to start moving in the behaviour test (mean = 11.11 ± 4.41 sec, Table 3), compared to individuals in simpler conditions (mean = 15.94 ± 3.52 sec, Table 3). Individuals housed in more complex conditions also had shorter latencies to find the entrance after they had started to move (mean = 193.66 ± 27.78 sec, Table 3), than individuals housed in simpler conditions (mean = 241.72 ± 22.79 sec, Table 3). Furthermore, individuals housed in more complex condition spent less time in the zone closest to their pen mates (‘Inner Circle’, mean = 68.83 ± 3.76 sec, Table 3), compared to individuals housed in simpler conditions (mean = 78.86 ± 2.55, Table 3) and were more likely to enter the zone without visual contact with pen mates (’Behind screen’, no = 12, yes = 23, Table 3), compared to individuals housed in simpler conditions (no = 24, yes = 12). Environmental complexity did not influence any of the brain monoamines examined (Table 4).

### Relationship between brain monoamines and altered response in a judgement bias test

How individuals changed their response from the first to the second judgement bias test was related to differences in dopamine turnover rate in mesencephalon; individuals with less altered response had higher dopamine turnover rate (Tables 1 and 2). No other clear relationships were observed between any of the other brain monoamines and behaviour in our multivariate behaviour assays and individual change in latency to colour cues in the judgement bias tests (Tables 1 and 2).

## Discussion

We have here shown that our treatments, early cold stress or housing conditions, did not have strong influence on behaviour on their own, but importantly that both in interaction with exposure to additional stressors had an additive effect and affected different behavioural responses of young female domestic fowl. Variation in optimistic bias was related to turnover rates of dopamine in the brain of these individuals. Thus, judgement bias in young female domestic fowl was influenced by both external and internal factors. These results will be discussed in turn below.

In our study, exposure to early cold stress in newly hatched chicks did not affect behavioural responses on its own. Interestingly, after chicks were exposed to additional stress, birds that were earlier cold stressed had shorter latencies to start moving in a multivariate behavioural test than did individuals that had only been exposed to the unpredictable stressors. Thus, we observe an effect of additive stress. Exposure of multiple stressors has previously been shown to have negative impact on fitness^50^, suggesting that additive exposure to stress can be detrimental. Here we observed a difference in a response to a novel environment. Movement in a novel environment likely entails a trade-off between motivation to reinstate social contact, motivation to explore the environment and motivation to avoid detection by potential predators^51^. In a previous study on domestic young chicks, it was concluded that young female chicks have a higher motivation for social reinstatement than males in a novel environment^52^, and a short latency to leave a start box has previously been linked to stress in chickens^51^. This in turn suggests that a short latency to move for our young female chicks most likely reflects a high motivation for social reinstatement. Stressed domestic chicks that were more motivated for social reinstatement also showed stronger fear responses by remaining longer in tonic immobility than unstressed chicks^53^. High motivation for social reinstatement may thus reflect a fear-reducing effect in the vicinity of conspecifics^53^.

In our study, we did not find support for judgement bias being influenced by our early cold stress treatment. The effect of stress on judgement bias in animals has received some attention in the last couple of years, and the effect varies between studies. For example, unpredictable chronic stress induced long-term negative effects on judgement bias in rats^33,54^, and lambs (*Ovis aries*^18^), but failed to do so in sheep^17^. Similarly, short-term stress induced pessimistic bias after stressful events like disbudding^19^, and maternal separation in dairy calves (*Bos taurus*^20^). However, optimistic bias increased in sheep after shearing^22^, and after restraint and social isolation^21^. Arguably, the timing, type, duration and intensity of the stressor are important for biasing cognitive processes.

We observed no clear differences in behavioural responses to the multivariate behavioural assays between individuals housed in more complex environmental conditions and those housed in simpler conditions before the unpredictable stressor. One possible explanation is that the difference in complexity was not pronounced enough to cause a difference between the two groups. Nonetheless, after exposing the chicks to unpredictable stress there was a clear difference between individuals housed in more complex versus simpler conditions. Individuals raised in a more complex environment had shorter latencies to start moving and to solve the detour than individuals raised in simpler environments. They also spent less time close to their conspecifics and tended to explore parts of the arena where they did not have visual contact with them, compared to individuals raised in simpler environments. Individuals housed in the more complex environmental treatment might therefore be described as more explorative and risk taking. Similar to our findings, domestic chicks dispersed more if they had early experience with objects that they could move out of sight from than if they did not have this experience^55^. Together, these findings suggest that both positive and negative experiences early in life interact to affect later behavioural responses and that multiple exposures to stress can cause changes in behaviour.

In our first judgement bias task, chicks approached the ambiguous cue more often than what we would expect if they had a neutral, or overall negative bias^9,27^. We interpret the high initial probability of approach as revealing an optimistic bias in chickens with similarities to what is observed in humans^39,40^. We further show that environmental complexity can help maintain this optimistic bias after exposure to stress. Even so, in accordance with a previous study on chickens^24^, we found no initial difference in judgement bias between the simpler and more complex conditions (i.e. before exposure to an additional stress). This was unexpected because a number of studies have shown increased optimistic bias in animals housed in enriched conditions compared to either barren or standard conditions (e.g. refs^12,26,27,48,49^, but see refs^24,56^). Perhaps under the conditions in our study even the complex environment might still have been relatively barren compared to nature. In addition, many of the other studies have used a within-individual design adding a component of past versus current conditions^12,27,48,49^, or have added aspects of unpredictability to the simpler condition^26^. Thus, the difference between our two environments was maybe not large enough for effects to be apparent without exposure of an additional stressor. The pens used in the simpler condition were not barren, but had substrate on the floor in which the chicks could perform dust-bathing behaviour, arguably a highly motivated behaviour in chickens (for review, see ref.^57^). Depriving chicks of dust-bathing opportunities may have led to more frustration and stress than providing this material and might explain the lack of a difference before the unpredictable stress. Interestingly though, environmental complexity seemed to act as a buffer against the added stressors. Specifically, chicks housed in a more complex environment remarkably remained optimistic after exposure to a period of severe unpredictable stressors, while chicks in simpler conditions became less optimistic after this stress exposure. Effects of environmental enrichment have been studied extensively (see ref.^58^ for review), including its effect on brain function and behaviour (e.g. refs^59–61^). Environmental enrichment has been shown to reduce negative effects of stress (e.g. refs^46,47^), for example by decreasing stress-induced changes in feeding behaviour, exploration^46^ and anxiety^47^. Further, we have recently shown that early cognitive stimulation can affect adult personality^45^ and previous studies have shown that environmental enrichment can induce optimistic bias (e.g. refs^12,26,27,48,49^). Nonetheless, we are not aware of any study that has shown that environmental complexity can help sustain optimistic bias after stressful events. It has been suggested that mood is adaptively regulating the threshold for positive and negative responses by taking into account the individuals physical state and the state of the environment^62^. It might thus be adaptive to alter future expectations of positive and negative events after experiencing aversive events during development in a suboptimal environment. It has been suggested that an increased expectation of negative events is indicative of anxiety whereas a decreased expectation of positive events indicates depression^63^. Here, there was a difference between individuals housed in simpler versus more complex environments in latency to approach the unrewarded negative cue and the close to negative cue, thus possibly indicating an increased level of anxiety in birds raised in simpler environments. Although, because there was a difference also toward the close to positive cue, chicks housed in simpler conditions could potentially be in a depressed state. Further, we observed a difference between individuals housed in simpler versus more complex environments in response to one of the previously learnt cues. This has been observed in previous studies^42,62^, but as far as we know, there are no explanations to why the treatments have caused a difference also to trained cues. It is possible that individuals that are in a negative affective state will also have a lower motivation to approach cues. For example, in humans it has been observed that approach motivation is linked to depression^64^, and depression is known to associate with negative judgement^1,2^. We therefore encourage future research on the link between depression, motivation and biased judgement in animals.

The nervous system controls complex behaviour through the influences of brain monoamines on behavioural states^65^. The brain monoamines, norepinephrine, serotonin and dopamine, are known to be related to emotional processing^40,66^. However, the neuroendocrine mechanisms underlying judgement bias remain poorly understood. Recent studies in humans suggest that enhancing dopaminergic function increases optimistic bias^39,40^. Similarly, recent studies on rodents suggest a possible role for dopamine^41,67^, serotonin^67,68^, and norepinephrine^67,69^ in biased judgement in animals. In accordance with these findings we demonstrated that dopamine activity in the brain is associated with optimistic bias in chicks. Dopamine is an important monoamine in reward learning and reward-seeking behaviour^70,71^. Further, recent findings show that dopamine is related to an enhanced feeling of pleasure when thinking about positive future events^39^, and that the ability to update negative information is impaired by dopamine^40^, which could help explain the differences in judgement that we observe.

The role of norepinephrine as well as serotonin in judgement bias remains more obscure. Studies either found no effect of drugs, independent on dose^41^, reported dose dependent responses^67^, or observed effects only on one type of response (i.e. increase or decrease in either positive or negative responses^67–69^). Norepinephrine and serotonin were not strongly associated with optimistic bias in the current study. Norepinephrine is involved in a variety of cognitive processes (e.g. perception, learning and memory) through its control of variation in arousal^72,73^, and serotonin is known as a complex neurotransmitter involved in many aspects of brain function and behaviour as well as being suggested to be an opponent partner to dopamine^74^. It has been proposed that dopamine promotes behavioural stimulation to seek rewards, while serotonin will inhibit stimulation when punishment could occur^74^. Indeed, because affective states range on a scale from optimistic to pessimistic, more than one system is probably involved in regulating affective states. More studies are therefore needed to better understand the role of monoaminergic systems in judgement bias control.

## Conclusion

The results from the current study demonstrate that additive stress can have a negative influence on behaviour and cognitive processes in domestic chicks, and that environmental complexity can buffer against these negative effects. Further, we show that dopamine activity in the brain is related to judgement biases in young female chickens. Taken together, these results improve our understanding of variation in behaviour and cognition, and highlight factors influencing variation in judgement bias in animals.

## Method

### Animals, housing and treatments

**Figure 2:**
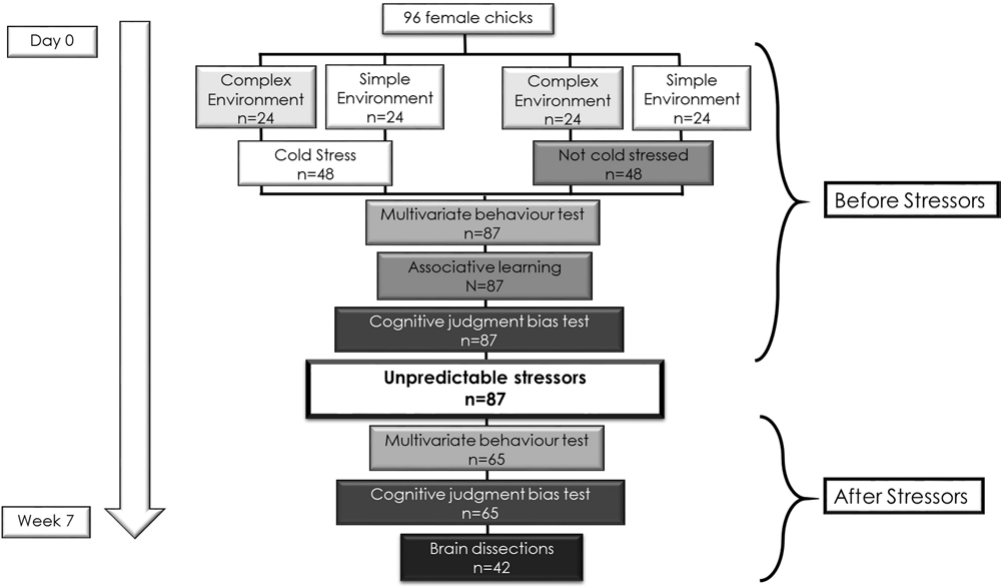
Schematic of the test procedure. See main text for further details.

All experiments were conducted at The Swedish Livestock Research Centre Lövsta (Swedish university of agricultural sciences, Uppsala, Sweden), June - July 2014. Female Bovans Robust chicks (n = 96), which are a laying breed of domesticated fowl, were collected at day 0 from a hatchery and transported in a ventilated car to the research facility. All chicks were kept in groups of 12 individuals in eight, 1.2 m^2^ pens. At arrival, chicks were divided into groups that were balanced according to their weight ensuring that all groups had similar composition of individuals. The chicks were raised without mothers to reduce potential maternal influences. All chicks had ad libitum access to commercial poultry food and water. To facilitate identification, chicks were initially individually marked with leg-rings, and at around 2 weeks with round, laminated paper tags (3 cm of diameter) in both wings. All tests (see Fig. 2 for schematic of the test procedure) were performed during the light hours of the day when birds are active, 9.00–18.00. Standard fluorescent lamps were used as light source during the tests. All observers were blind to treatments and results in the preceding tests.

The chicks were assigned to 4 treatment groups in a two by two set up (separated into 8 pens, Figure S1). The pens where divided into two blocks of 4 pens in which treatments were semi-randomly distributed in the room (see Figure S1). Half of the chicks (four pens) were raised in more complex environmental conditions (‘complex environment’) whereas the other half (four pens) were raised in simpler environmental conditions (‘simple environment’). Pens that were categorised as complex had wood shavings, a round pellet feeder, a water bell and wooden blocks on the floor to encourage movement and exploration (see Figure S2). These pens had perches at different heights underneath a sheltering roof, as well as a secluded and protected area under a lower shelf with cloth strips hanging in front of the entrance. Chicks raised under simpler environmental conditions had wood shavings on the floor, a pellet feeder and a water bell, but the pen was otherwise lacking any additional interior or shelter (Figure S2).

Two days post-hatching, chicks from two randomly selected complex and two randomly selected simple pens were exposed to a substantial decrease in room temperature over a period of 6 hours (‘cold stressed’), while chicks from the remaining pens (two complex and two simple) were kept in optimal temperature (‘not cold stressed’). All chicks were placed in pairs in small compartments (20 W × 15 L × 15 H cm) in boxes (box size 40 W × 60 L × 15 H cm) and were left alone. For the cold stress treatment, the boxes were placed directly in a room where the temperature was 18–20 °C, whereas for the unstressed treatment groups the boxes were placed in a room at the optimal 33 °C. The first 10 days after hatching the thermoregulation in chicks is underdeveloped^75^, making them especially vulnerable to cold stress^76^. The temperature used as cold stress here was decided by pilot studies prior to the experiment. The average body temperature of the cold stressed chicks decreased by 2.9 °C whereas it only decreased by 0.8 °C for the unstressed chicks, supporting the effectiveness of this treatment as a stressor.

At 4 weeks of age, we exposed all chicks (both cold stressed and those not stressed) to a battery of stressors that were chosen to stimulate an unpredictable environment. Unpredictability and lack of control are major sources of stress that have been well described, especially in rodents (reviewed by^77^). Unpredictability has also been used as a stressor in chickens, causing learning difficulties^78^, long-term effects on behaviour^79^ and even epigenetic effects on behaviour in offspring of stressed parents^79,80^. Similarly, acute noise exposure above 80 dB increased corticosterone levels in broiler chickens already after 10 minutes^81^. The unpredictability included stressors that are common in husbandry practices such as novel water bells, extra feeders, cleaning of pens and higher levels of human entrance to the facility. In addition, these stressors were used: (i) heavy metal music playing at 90 dB during 1 hour, at different times of day, 5 days in a row, (ii) unpredictable noise at 90 dB in random intervals during the day (including sounds of predators and other animals, and mechanical noise such as trains, airplanes and ambulances), and (iii) lights going on and off unpredictably during both day and night the last three days of the stress treatment. From behavioural observations during this week it was found that feeding behaviour was almost eliminated during the hour that the heavy metal music was being played, supporting the effectiveness of this treatment as a stressor.

### Judgement bias test

At 2 weeks of age, chicks were exposed to a judgement bias test. Chicks were collected from their home pen and brought to a test room in a small transport box. The temperature in the test room was kept at the same temperature as the home pen.

After initial pre-training where chicks were familiarised with the test arenas (plastic boxes covered with corrugated cardboard paper, measuring 46 W × 68 L × 42 H cm), rewards (mealworms) and handling^45,82^, chicks were singly trained to associate a colour cue (black or white, n_white_ = 46, n_black_ = 49) with a reward (1/3 of a mealworm). The colour cues were printed on photo paper and laminated. Colour signs (one black and one white, 9 × 9 cm) and similarly coloured bowls were placed on both sides of a small partition (Figure S3a). The reward was placed close to the front edge of the bowls to make sure that the chicks could not see the reward before making their choice. During training, chicks had to make an active choice between the two colour cues (black and white) that were presented to them (for further details see ref.^45^). Chicks were allowed to make an unrestricted amount of choices during each training session and were allowed three sessions that each lasted ca. 15 minutes and were at least 1 hour apart. Chicks needed to reach a criterion of 6 correct choices in a row, and to again reach this criterion on the following day before proceeding to the judgement bias test. In the judgement bias test we presented colour cues one by one to the chicks in the middle of the arena (the partition had been removed, see Figure S3b). In addition to the previously rewarded and unrewarded colour cues we introduced three ambiguous colour cues that were intermediate between the previously rewarded and unrewarded colour cues; dark grey: 25% white/75% black, medium grey: 50% white/50% black, and light grey: 75% white/25% black. Latency (sec) until the chick had approached the colour cue after being released was recorded as a behavioural measure of the chicks’ response to these ambiguous cues. A short latency to an ambiguous colour cue similar to that chick’s unrewarded colour cue would indicate an optimistic chick, whereas a long latency to an ambiguous colour cue similar to that chick’s rewarded colour would indicate a less optimistic chick (sensu e.g. refs^3,7,26^). During testing, the chick was placed in the arena with her head facing away from the colour cue to prevent the chick from making any decision before starting to move. A maximum latency of 30 seconds was allowed to approach the colour cue. If the chick did not approach the presented stimuli within this time, the trial was aborted and the next trial started. Similarly, if the chick jumped out of the arena, the trial was aborted and the chick received a max score of 30 seconds and the next trial started. The order of the colour cues was presented according to a pre-determined, semi-randomized schedule, where two of each intermediate grey colour cue were presented in-between eight rewarded and seven unrewarded cues. Therefore, any potential order effect should not systematically have affected any specific cue. To keep the chicks motivated throughout the test we continued to reward the colour that the chicks had been trained to associate with a reward, while all other colours were left unrewarded.

A second judgement bias test was performed when the chicks were 5 weeks old and after the chicks had been exposed to the battery of unpredictable stressors. To make sure that the association between colour cue and reward remained, we performed a new associate learning trial prior to the test. Again the chicks were required to have 6 correct choices in a row before proceeding to the judgement bias test. The judgement bias test was performed in a similar fashion as the first test, the only differences being that the arenas were larger during the second test (measuring 49 W × 99 L × 50 H cm) as the chicks had grown in size. Sixty-five chicks were tested in the second judgement bias test (9 chicks failed the associative learning task and 22 chicks died, most likely as a consequence of contaminated blood used in an immunological test, not part of this study) performed on the chicks after the first judgement bias test.

### Multivariate behavioural assay

Birds were exposed to a multivariate behaviour assay at 2 and 5 week of age (i.e. before and after exposure to unpredictable stress, and before each judgement bias tests). The test was modified from a previously used test^83^. The arena was adjusted between the two test occasions to compensate for the increased size of the birds. In both trials the arena had a start box (40 W × 40 L × 40 H) that included a detour; a u-shaped route from the starting position leading to the larger arena (Figure S4a). The larger arena was a circular arena (first test: Ø 1.20 m, second test: Ø 1.60 m, Figure S4a,b) with a companion box (first test: Ø 0.30 m, second test: Ø 0.40 m, Figure S4a) out of wire mesh in the centre. The arena was built of aluminium plate with a wooden floor. Four cardboard screens (first test: 0.20 W × 0.20 H m, second test: 0.30 W × 0.30 H) were mounted inside the arena opposite to each other, creating a not fully closed circle measuring (first test: Ø 0.70 m, second test: Ø 0.80 m), aiming to encourage birds to be risk-taking and explore the arena. The arena was divided into 3 zones, which were not visible to the chicks, but that were used to observe their use of the arena. In the ‘Inner circle’ the chicks were close to their conspecifics, in the ‘Outer circle’ they were not in close proximity to conspecifics but could still see them, and in the ‘Behind screen’, they were not in close proximity to conspecifics and had no visual contact with them (Figure S4). A chick was considered to be in a particular zone when at least 50% of its body was located in the zone.

Chicks were calmly collected in groups of 4 directly from their home pen. All four chicks were placed in the companion box and left there for 3 min to habituate. Thereafter, one of the birds, the focal bird, was placed in the start box in the periphery of the arena (Figure S4a). If the chick did not leave the starting box within 7 minutes i.e. did not solve the detour, the barrier was removed and the test continued, discarding the detour part of the test. All focal chicks received 5 minutes to move freely in the area, time starting as soon as a chick entered the arena after the detour. Thus, all chicks received an equal amount of time to explore the arena despite differences in initial fear response or problem solving skills (i.e. despite differences in latency to move and latency to detour). Behaviours scored (in seconds) were; ‘latency to start moving’, ‘latency to find the entrance’ (i.e. latency to detour and cross the line of the detour barrier). We also video recorded ‘proportion of time spent in the ‘Inner circle’ and the ‘Outer circle’ as well as if they explored the ‘Behind screen’ or not (Figure S4b). Videos were decoded blindly by the same observer (IC). After a chick had completed the test, it was returned to the companion box and a new bird was tested.

### Analyses of brain monoamines

Brains of birds that successfully finished the second judgement bias test (n = 65) were sampled at 7 weeks of age. The chicks were anesthetized by a hard hit to the head and euthanized by swift cervical decapitation (in accordance with the ethical permit approved for the study). Immediately following euthanasia, brains were removed and dissected into 7 parts^84^ on a metal tray chilled with dry ice. The left mesencephalon, telencephalon, hypothalamus-thalamus and optic tectum were snap frozen on dry ice within a few minutes from euthanasia (mean = 2.5 min, range = 1.42–5.25 min). The brain tissues were subsequently stored in −80 °C until they were analysed.

Frozen tissue samples were weighed before being homogenised in 1500 μl, 4% (w/v) ice-cold perchloric acid containing 100 ng/ml dihydroxybenzylamine (DHBA). DHBA was used as an internal control to correct for potential degradation. Homogenised and thawed samples were centrifuged at 15 000 rpm for 10 min at 4 °C. The supernatant was used for high performance liquid chromatography with electrochemical detection (HPLC-EC) as described in detail by^85^. Briefly, the HPLC-EC system consisted of a solvent delivery system model 582 (ESA, Bedford, MA, USA), an autoinjector Midas type 830 (Spark Holland, Emmen, the Netherlands), a reverse phase column (Reprosil-Pur C18-AQ 3 μm, 100 mm × 4 mm column, Dr. Maisch HPLC GmbH, Ammerbuch-Entringen, Germany) kept at 40 °C, and an ESA 5200 Coulochem II EC detector (ESA, Bedford, MA, USA) with two electrodes with reducing and oxidizing potentials of −40 mV and +320 mV. A protecting electrode with a potential of +450 mV was used before the analytical electrodes to oxidize any contaminants. The mobile phase had a flow of 1 ml/min and was prepared by adding 75 mM sodium phosphate, 1.4 mM sodium octyl sulphate and 10 mM EDTA, and around 7% acetonitrile to deionized water that was brought to pH 3.1 with phosphoric acid. Norepinephrine (NE), dopamine (DA), serotonin (5-HT), and the metabolite of dopamine; 3.4-dihydroxyphenylacetic acid (DOPAC) and the metabolite of serotonin; 5-hydroxyindoleacetic acid (5-HIAA), were analysed. The concentration of brain monoamines and metabolites were calculated from a reference curve made using standards. Concentrations were estimated as nanogram per gram brain tissue. The ratios of DOPAC/DA and 5-HIAA/5-HT were calculated and used as an index of dopaminergic and serotonergic activity, respectively.

### Ethical note

In this study, we only use brains of individuals that reached our learning criteria for associative learning, and thus started with a relatively large number of birds, to ensure a sufficient sample size for our statistical analysis. Further motivating our relatively large number of animals used, all individuals were also part of an additional study. The experiment was conducted according to ethical requirements in Sweden, and approved by Uppsala Ethical committee (ethical permit number C70/14).

### Statistical analyses

All statistical analyses and model selections were conducted in R version (3.2.3).

#### Analyses of variation in latencies obtained in judgement bias tests

The two first colour cues in our judgement bias test were the rewarded colour cue followed by the unrewarded colour cue. Data from these trials were not included in the statistical analysis since they were considered to be acclimatisation trials to the new test set-up.

To explore whether cold stress and environmental complexity affected latencies in the first judgement bias test, we performed Mann-Whitney U-tests for each colour cue. We adjusted for multiple comparisons using Bonferroni correction.

Because we observed no difference between the treatment groups in the first judgement bias test (see statistics above) we continued by exploring whether there was variation in the changed response between the fist and the second judgement bias test that was explained by our treatments or influenced by brain monoamines. The change in latency ‘changed latency’ was calculated as (latency to colour cues in test 1 – latency to colour cues in test 2, +30 to allow all values to be on a positive scale). This variable had a normal distribution and parametric tests were used. We constructed full linear mixed-effects models containing our predictor variables; environmental complexity (‘complex environment’ vs. ‘simple environment’); cold stress (‘cold stressed’ vs. ‘not stressed’); interaction between environmental complexity and cold stress; brain monoamines (‘NE’ for norepinephrine, ‘DA’ for dopamine, ‘DOPAC/DA’ for dopamine turnover rates, ‘5-HT’ for serotonin, and ‘5-HIAA/5-HT’ for serotonin turnover rates), in each brain area measured (‘Tel’ for telencephalon, ‘Ht’ for hypothalamus-thalamus, ‘Mes’ for mesencephalon, or ‘Ot’ for optic tectum), and ‘Cue’ to account for the inclusion of the 5 colour cues used in judgement bias tests. We used separate models containing treatment and one of the brain monoamines at the time, due to co-linearity of monoamines within brain areas. In all models, ‘pen’ and bird ‘ID’ were included as cross-random effects. In cases where the monoaminergic activity had a significant effect on the response in the judgement bias tests, we explored whether increased monoamine levels or increased metabolites explained the increased activity by performing Spearman rank correlations between the monoamine and behavioural response, and between the metabolite and the behavioural response.

Our general approach was to use classical statistics with p-values, however when there were many covariates we tried to minimise the number of models by using model selection. We used the R-package ‘MuMIn’ and the function ‘dredge’ to determine models which best explained variation in ‘changed latency’. To estimate the most parsimonious models we used Akaike’s Information Criterion (AICc), with the conventional cut-off point of delta 2 (ΔAICc <2 change from the best ranking model^86,87^), for models to be considered (all models within ΔAICc <2 are shown in the results section below). Models were ranked according to their AICc value (AIC values adjusted for small sample sizes) and weight (ω), where lower AICc values and higher ω suggest a better goodness of fit^86^. AICω was used to evaluate the relative support for the best models, and sum of AICω (ΣAICω) used to evaluate the relative support for the individual variables in the models. Sum of AICω were obtained for all models within cumulative weight 0.95. For comparison, we included ‘Null models’ containing only the random effects. If the null model was included in ΔAICc < 2, the model was interpreted as uninformative because it did not explain the variance of the data better than a null model. Random effects were estimated using intra class correlations from the R package ‘multilevel’ (Table S1).

Environmental condition explained changes in latencies toward the cues between the two judgement bias tests (see results above). To analyse whether this difference was due to changed latencies in the complex or simple condition and to see at what cue the difference was observed, we performed Wilcoxon Signed Rank Test on mean latencies for each individual for each cue and adjusted for multiple comparisons with Bonferroni correction. We used a non-parametric test because the raw latency data was not normally distributed.

#### Analyses of variables from the multivariate behavioural tests

Because the assumption of normality was not met for the behavioural variables attained from the multivariate behavioural assay, the effects of stress and environmental complexity were analysed using generalized mixed linear models. If models were over-dispersed, we included an observation-level random intercept to account for the extra variance in the residuals. Latency to start moving was subtracted from latency to solve the detour to obtain independent measures. In the first test, there was no variation between individuals in time spent in the different zones (i.e. all individuals were in the zone close to the pen mates), therefore only variables from the second test were analysed. Because times spent in the ‘Behind screen’ zone in test 2 was zero inflated, we analyzed it as a binomial model investigating if animals explored the area ‘Behind screen’ or not.

#### Analyses of brain monoamine levels

To explore the effect of cold stress and environmental complexity on levels of brain monoamines, we constructed linear mixed-effects models with each monoamine as response variables and with cold stress, environmental complexity, and their interaction, as predictors. If the interaction term was non-significant, we re-ran the model without the interaction term. We included ‘pen’ as a random effect. Because the variables had non-normal distributions, we chose suitable transformations using box-cox. This resulted in log (x) for Mes NE, Mes 5-HT Tel DOPAC/DA, OT DA, √ (x) for Mes DA, Mes 5-HIAA/5HT, Ot DOPAC/DA, -1(X) for Tel 5-HIAA/5-HT, Ht NE, Ht DA, Ht 5-HT, Ht 5-HIAA/5-HT, 1000000/(X) ^ 3 for Ht DOPAC/DA and Ot 5-HIAA/5-HT.

## Electronic supplementary material

Supplementary Information
