# Supplementary material for: Environmental complexity buffers against stress-induced negative judgement bias in female chickens
Source: Sci Rep. 2018 Mar 29;8:5404. doi: 10.1038/s41598-018-23545-6 (PMC5876351; doi:10.1038/s41598-018-23545-6)
Supplement: Supplementary file 1 — Supplementary Information [file 41598_2018_23545_MOESM1_ESM.pdf]

Environmental complexity buffers against stress-induced negative judgement bias in female chickens

Josefina Zidar, Irene Campderrich, Emelie Jansson, Anette Wichman, Svante Winberg, Linda Keeling and Hanne Løvlie.

Supplementary material

The supplementary material contains:

Supplementary figures:

- Figure S1. Graphic illustration of arrangement of pens
- Figure S2. Graphic illustration of the complex pen
- Figure S3. Schematic of arena used in associative learning and judgement bias testing
- Figure S4. Pictures of the arena used in a multivariate behavioural test

Supplementary tables:

- Table S1. Intra class correlations of the random effects ‘Pen’ and ‘ID’.

Supplementary figures

Figure S1. Position of pens in the room according to treatment. NS = Not Cold Stressed, CS = Cold Stressed. A blue square with a grid indicate a complex pen. The pens were divided in two blocks so that treatments would be evenly distributed in the room. Within a block, pens were semi-randomly assigned to the treatments.

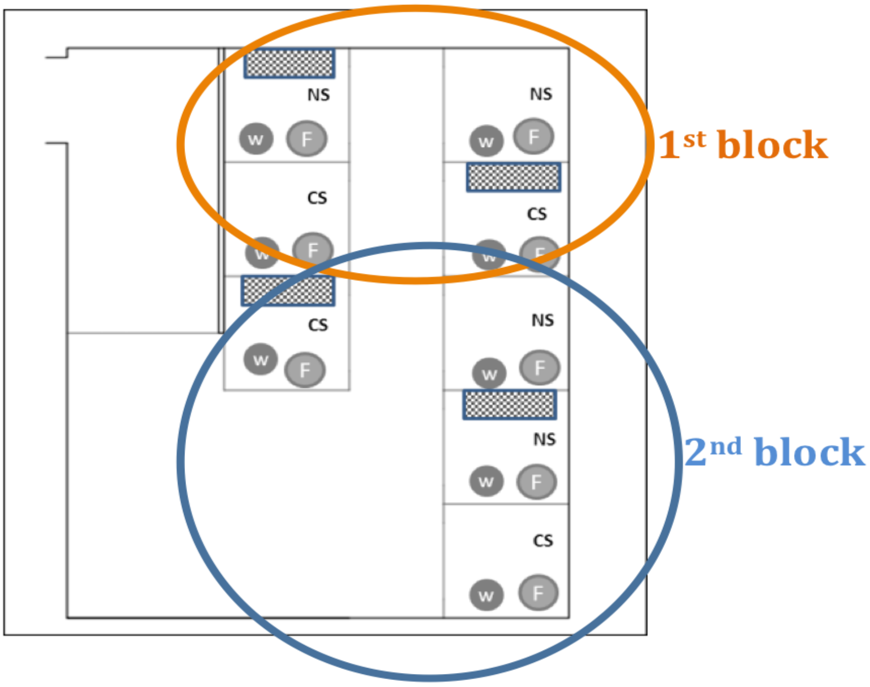

Figure S2. Drawing (not according to scale) of a) top view and b) side view of a complex pen that contain a round pellet feeder, a water bell, wooden blocks, perches and a secluded area under a sheltering roof. The simple pen only had a round pellet feeder and a water bell. Both pen types had wood shavings as substrate.

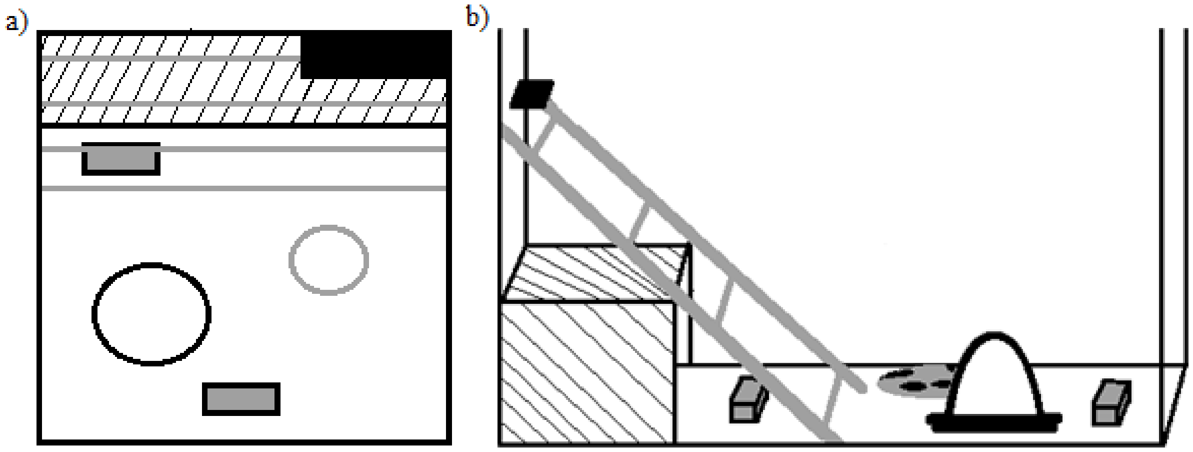

Figure S3. Schematic of the arena (not according to scale) used for a) training (associative learning) and b) testing, of female Bovans Robusta chicks in a cognitive judgement bias test. Chicks were first trained to associate a colour cue with a reward before their reactions to also intermediate cues were measured.

a)

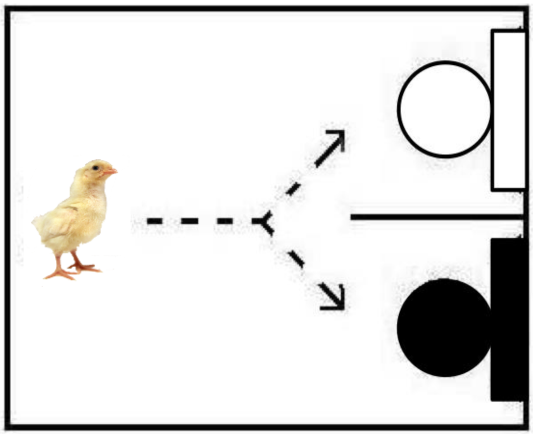

b)

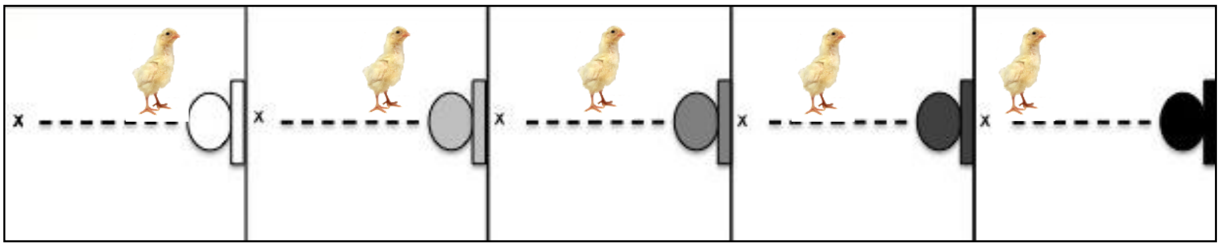

Figure S4. Arena for multivariate behaviour testing of domestic female chicks, showing a) 'Start box' (i.e. where chicks were released in the beginning of the test), 'Detour' (i.e. chicks had to make a detour to get to the entrance of the arena) and companion box (i.e. wire mesh box where 3 companion birds where held), cardboard screens encouraging exploration, and b) imaginary arena zones used to measure behaviour. The tree zones where, 'Inner circle' where chicks stayed close to conspecifics, 'Outer circle' where chicks spent time further away from conspecifics, but with visual contact, and 'Behind screen', where the chicks venture to an area away from conspecifics and without visual contact.

a) b)

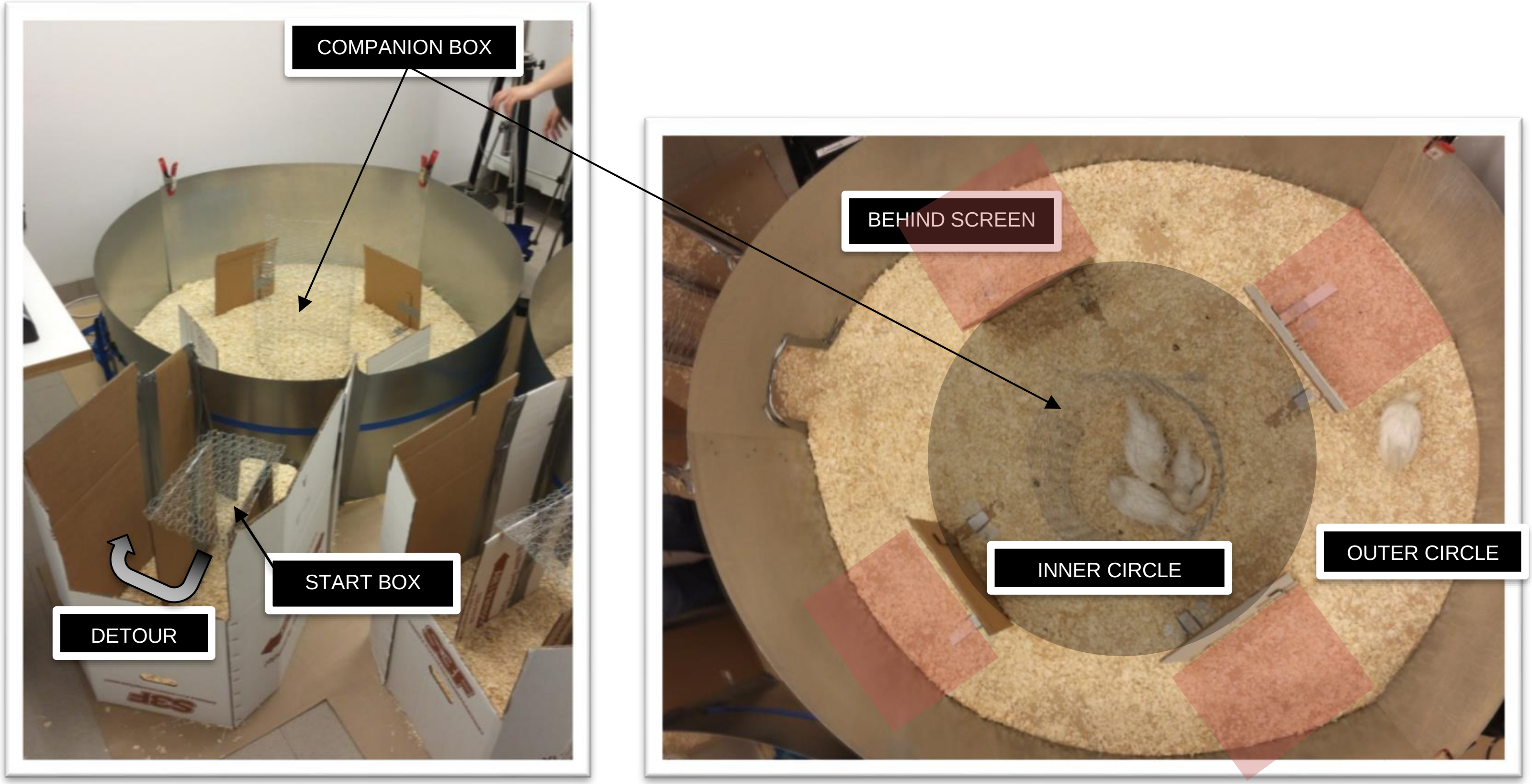

Supplementary tables

Table S1. Estimated intra-class correlation for random effects. Models for associative learning included 'Pen' as random effect and models for judgement bias included 'Pen' and 'ID'as random effects. The effects of 'Pen' and 'ID' were weak. 1st test refers to the multivariate behaviour test prior to the unpredictable stressors and the 2nd test to the multivariate behaviour test after the unpredictable stressors. See main text for further details on variables descriptions.

Response	Pen	ID
Latency to move (1st test)	0.04	-
Latency to find entrance (1st test)	0.02	-
Latency to move (2nd test)	0.01	-
Latency to find entrance (2nd test)	-0.07	-
Time in Inner Circle (2nd test)	-0.06	-
Learning speed	-0.13	-
Latency to cues	0.10	-0.005
